# Maternal and child factors associated with child body fatness in a Ghanaian cohort

**DOI:** 10.1017/S1368980019001745

**Published:** 2019-07-25

**Authors:** Sika M Kumordzie, Harriet Okronipa, Mary Arimond, Seth Adu-Afarwuah, Maku E Ocansey, Rebecca R Young, Helena J Bentil, Solace M Tamakloe, Brietta M Oaks, Kathryn G Dewey

**Affiliations:** 1 Program in International and Community Nutrition, Department of Nutrition, University of California, Davis, 3253 Meyer Hall, Davis, CA 95616, USA; 2 Intake – Center for Dietary Assessment, FHI 360, Washington, DC, USA; 3 Department of Nutrition and Food Science, University of Ghana, Legon, Ghana; 4 Department of Nutrition and Food Sciences, University of Rhode Island, Kingston, RI, USA

**Keywords:** Body composition, Diet, Factor analysis, Physical activity, Ghanaian children

## Abstract

**Objective:**

We aimed to identify factors (child diet, physical activity; maternal BMI) associated with body composition of Ghanaian pre-school children.

**Design:**

Longitudinal analysis of the International Lipid-Based Nutrient Supplements (iLiNS)-DYAD-Ghana randomized trial, which enrolled 1320 pregnant women at ≤20 weeks’ gestation and followed them and their infants until 6 and 18 months postpartum, respectively. At follow-up, child age 4–6 years, we collected data on body composition (by ^2^H dilution), physical activity and diet, extracted dietary patterns using factor analysis, and examined the association of children’s percentage body fat with maternal and child factors by regression analysis.

**Setting:**

Eastern Region, Ghana.

**Participants:**

Children 4–6 years of age.

**Results:**

The analysis included 889 children with percentage body fat and dietary data at follow-up. We identified two major dietary patterns, a snacking and a cooked foods pattern. Percentage body fat was positively associated (standardized *β* (se)) with maternal BMI at follow-up (0·10 (0·03); *P* = 0·003) and negatively associated with physical activity (−0·15 (0·05); *P* = 0·003, unadjusted for child gender), but not associated with the snacking (0·06 (0·03); *P* = 0·103) or cooked foods (−0·05 (0·07); *P* = 0·474) pattern. Boys were more active than girls (1470 *v.* 1314 mean vector magnitude counts/min; *P* < 0·0001) and had lower percentage body fat (13·8 *v.* 16·9 %; *P* < 0·0001).

**Conclusions:**

In this population, maternal overweight and child physical activity, especially among girls, may be key factors for addressing child overweight/obesity. We did not demonstrate a relationship between the dietary patterns and body fatness, which may be related to limitations of the dietary data available.

The prevalence of childhood overweight and obesity is increasing worldwide. The global age-standardized prevalence in children and adolescents increased from 0·7 % in 1975 to 5·6 % in 2016 among girls and from 0·9 % in 1975 to 7·8 % in 2016 among boys^(^^1^^)^. Although the prevalence of obesity is higher in higher-income than in lower-income countries, the latter experienced a greater increase in prevalence during that 42-year period, up to a 400 % increase in prevalence per decade in places such as Southern Africa compared with a 30–50 % rise in prevalence per decade in high-income regions^(^^1^^)^. This situation is of particular concern because the rates of undernutrition remain high in many lower-income countries despite the increase in overweight, so these populations are experiencing a ‘double burden’ of malnutrition.

There is evidence that overweight in childhood tends to persist into adulthood, although the strength of evidence is moderate^(^^2^^)^. This suggests that the increasing rates of childhood overweight/obesity may lead to higher rates in adulthood. Childhood obesity is associated with a range of serious health complications and an increased risk of premature illness and death in later life^(^^3^^)^. Obesity in adulthood is also known to increase the likelihood of developing CVD, type 2 diabetes and its associated retinal and renal complications, non-alcoholic fatty liver disease and other disorders^(^^4^^)^.

Diet and physical activity are modifiable factors associated with overweight/obesity. Several studies have reported associations between diet and body composition among children and adolescents^(^^5^^–^^7^^)^, although very few such studies have been conducted in low- and middle-income countries. In recent decades, dietary pattern analyses using statistical tools have been increasingly used to examine the relationship between diet and health. These dietary pattern studies take advantage of the collinearity between dietary variables to examine their collective influence instead of looking at individual nutrients or food groups^(^^8^^)^.

Ghana is one of many countries experiencing the double burden of malnutrition. According to the most recent (2014) Demographic and Health Survey (DHS), 11 % of children under the age of 5 years were underweight, 19 % were stunted and 3 % were overweight, and the prevalence of overweight/obesity among women was 40 %^(^^9^^)^. The rates of stunting, wasting and underweight were lower in the 2014 DHS compared with 2008. There was also a decrease in child overweight, from 5 to 3 %; however, this rate has remained between 3 and 5 % since the 2003 survey. On the other hand, overweight/obesity among women increased from 30 % in 2008 to 40 % in 2014.

Our objective was to determine whether child diet, child physical activity or maternal BMI was related to child body fatness in a cohort of pre-school children who had participated in the International Lipid-Based Nutrient Supplements (iLiNS)-DYAD trial in Ghana as infants^(^^10^^)^. This cohort was followed up at 4–6 years of age to examine long-term effects of supplementation on growth and body composition^(^^11^^)^, development^(^^12^^)^ and physical activity, among other outcomes. In the present paper we report results of an exploratory analysis of the factors related to body composition in the iLiNS-DYAD cohort. Because of the trend towards greater child overweight in low- and middle-income countries, it is important to identify such factors to design effective interventions to prevent excess body fatness.

## Methods

### Study design

Data for the current analysis were obtained from the iLiNS-DYAD-Ghana trial follow-up. Briefly, the parent iLiNS-DYAD trial was a partially double-blind randomized controlled trial that enrolled 1320 women aged 18 years or older at ≤20 weeks’ gestation attending antenatal clinics in four main health facilities in the Yilo and Lower Manya Krobo districts of the Eastern Region of Ghana. The women were randomized to one of three treatments: (i) daily iron and folic acid capsule (IFA) during pregnancy and daily tablet containing 200 mg calcium during the first 6 months postpartum and no infant supplementation; (ii) daily multiple micronutrient capsule, MMN (1–2 times the RDA of eighteen vitamins and minerals), during pregnancy and the first 6 months postpartum and no infant supplementation; or (iii) daily 20 g (474 kJ; 118 kcal) lipid-based nutrient supplement (LNS) during pregnancy and the first 6 months postpartum followed by infant LNS supplementation from 6 to 18 months of age. The iLiNS-DYAD trial was partially double-blind because IFA and MMN capsules were colour-coded by an independent team and no investigator, study worker or participant knew the identity of the capsules except by colour. However, because of the obvious difference between the capsules and the LNS supplement it was not possible to completely blind the fieldworkers and study participants. Details of the trial and supplement composition are published elsewhere^(^^13^^)^.

**Fig. 1:**
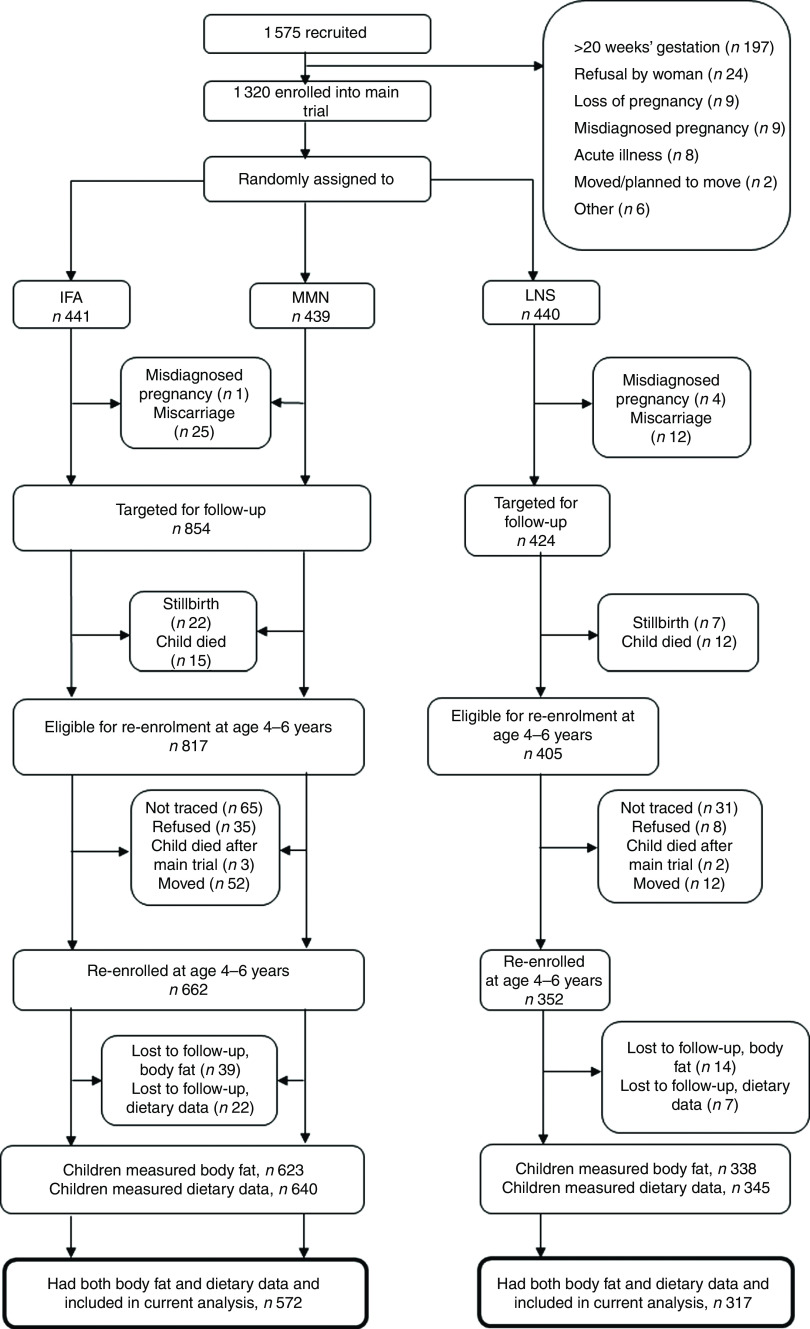
Study profile of the International Lipid-Based Nutrient Supplements (iLiNS)-DYAD-Ghana trial (IFA, iron–folic acid; MMN, multiple micronutrients; LNS, lipid-based nutrient supplement)

The follow-up study occurred when children were 4–6 years of age. The protocol for the trial (clinicaltrials.gov, NCT00970866) was updated to include the outcomes assessed during the follow-up, which included body composition. All children who were born to the randomized pregnant women in the main trial and were alive at the time of follow-up were eligible to participate (study flow shown in Fig. 1). Participants were contacted using the last known address and contact number provided. Field staff called to set up an appointment and visited the caregiver to obtain informed consent for the follow-up activities. After consent had been given, participants were scheduled for the data collection visits. The outcome in the analysis was child body fatness. Child factors (dietary patterns, an estimate of physical activity and school attendance) and maternal factors (current BMI, age, education, nulliparity at enrolment into the trial and household asset score) were assessed as potential predictors of child body fatness.

### Data collection procedures

Anthropometric measurements of the women and children were taken according to WHO standard procedures^(^^14^^)^ by trained anthropometrists. Height was measured using a stadiometer (Seca 217) to 0·1 cm and weight to the nearest 50 g (Seca 875 scale). All measurements were taken in duplicate and in triplicate if the first two measurements differed by a predefined amount: 0·1 kg for weight and 0·5 cm for height. We computed weight-for-age and height-for-age *Z*-scores using the WHO Anthro software macros in the statistical software package SAS version 9.4.

Body fat of the child was estimated using the ^2^H dilution method for total body water measurement^(^^15^^)^. Details of equilibration time determination are published elsewhere^(^^11^^)^. Briefly, each child was administered a ^2^H dose based on body weight. Saliva samples were collected 2·5 and 3 h after dose administration. These samples were analysed for ^2^H enrichment from which total body water was calculated. Fat-free mass was estimated from total body water by a standard equation. The difference between body weight and fat-free mass is fat mass, which was expressed as a percentage of body weight (percentage body fat).

Physical activity was estimated in a random sub-sample of 353 children, using the ActiGraph wGT3X-BT triaxial accelerometer over a 1-week period (ActiGraph GT3X, Pensacola, FL, USA). Accelerometers were fitted to an elastic belt and fastened to the child’s right hip by trained data collectors. Caregivers were instructed to let the child wear the accelerometer continuously day and night unless they experienced discomfort. Activity was stored in 60 s intervals or epochs. Physical activity data were analysed using ActiLife data analysis software version 6.13.1 to generate mean vector magnitude accelerometer counts/60 s per participant. Vector magnitude is the outcome of the accelerometer device and the counts represent the intensity of bodily movements. The percentage of time spent in moderate/vigorous physical activity was also determined using the cut-offs proposed by Evenson *et al.*^(^^16^^)^.

Dietary data were obtained from caregiver report using an FFQ primarily designed to capture sugar-sweetened beverages, sweet and savoury snacks, as well as food groups usually consumed during meals. The questionnaire was administered to the caregiver by a trained fieldworker, who recorded how many times in the 7 d preceding the interview each item in a list of different food/beverage items was consumed by the child. There were thirty-one items (dietary variables) on the questionnaire which grouped related foods/beverages such as fruit or flavoured drinks, chocolate or malt drinks, etc. as one item. The reported number of times consumed represented the score for that dietary variable. Details of the questionnaire design and method used to collect the dietary data are described elsewhere^(^^17^^)^.

The Open Data Kit (ODK) software version 1.4.7^(^^18^^)^ was used for data collection and all questionnaires were programmed on tablets. The ODK system had quality checks built in to minimize entry of implausible values. Further data cleaning was done by the data manager and supervisors in the field by running SAS codes to check the data.

### Sample size and data analysis

As reported elsewhere, data on percentage body fat at the 4–6 years follow-up were available for 929 children^(^^11^^)^ and dietary data were available for 985. The relationship between physical activity and percentage body fat was examined in a sub-sample of children who were selected for the physical activity assessments (*n*353). With these sample sizes, we had 80 % power to detect a correlation of ≥0·09 between percentage body fat and the dietary variables and a correlation of ≥0·15 between percentage body fat and physical activity.

**Fig. 2:**
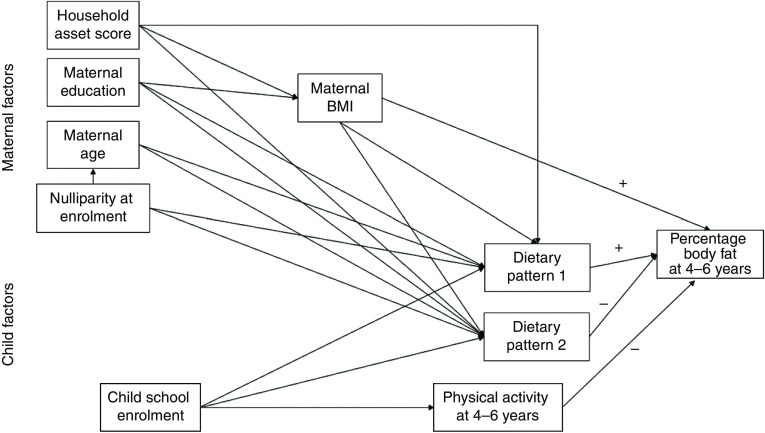
Conceptual model of the relationships of maternal and child factors with child percentage body fat

The conceptual model for the current analysis is shown in Fig. 2. The main outcome was percentage body fat at 4–6 years. Factors examined for their association with the main outcome were child physical activity, maternal BMI at follow-up and the child dietary patterns. We examined maternal education and household asset score at follow-up as factors potentially related to maternal BMI. We also examined factors potentially related to the dietary patterns, including maternal BMI and age at follow-up, maternal education at follow-up, household asset score at follow-up, nulliparity at enrolment and child school enrolment (yes/no). Household asset score was constructed based on ownership of a set of assets (radio, television, refrigerator and stove), lighting source, drinking-water supply, sanitation facilities and flooring materials, developed into an index (with a mean of 0 and an sd of 1) using principal components analysis^(^^19^^)^. We also collected information on maternal education and child school enrolment.

Data analysis was done using SAS version 9.4. All tests were considered significant at *P* < 0·05.

To identify the dietary patterns, factor analysis was employed in SAS using the thirty-one dietary variables. Our first analysis was exploratory, allowing the factor analysis to retain as many factors (dietary patterns) as possible. The scree plot obtained from this initial analysis levelled off at four factors, therefore we retained four factors in subsequent analysis. Of the four factors obtained, two of the factors accounted for 80 % of the variance in the data and the two factors also had eigenvalues greater than the recommended 1·25 (eigenvalue for factor 1 = 2·71; factor 2 = 1·60)^(^^20^^)^ and are the dietary patterns included the present analysis. The factors were rotated using the varimax method. The dietary patterns were described using the dietary variables that had an absolute factor loading of at least 0·35. Each respondent received a score for each of the two factors retained and the scored variables were our dietary pattern variables.

Next we examined the relationship for each path in the conceptual model (Fig. 2) between factor–outcome and factor–factor using regression analysis. For each regression model, we adjusted for child age (minimally adjusted model) and additionally for pre-specified covariates (child sex and intervention group) in fully adjusted models. Because outliers can strongly influence *β* values, we examined each regression model for observations with too much leverage. Observations (either *x* or *y* variables) with a high leverage (>0·02) were truncated to the 2·5th or 97·5th percentile. For all predictive models, the linearity assumption was tested by performing normality testing on the residuals and plotting the residuals *v*. fit scatterplot. All variables were normally distributed except dietary pattern 2, which had a bimodal distribution and was categorized as <median or ≥median. To avoid multicollinearity, any covariate–factor or factor–factor relationships with an absolute correlation coefficient of ≥0·75 were not included in the final model. All continuous variables were standardized to the mean and sd of the cohort before the regression analysis.

## Results

The children included in the present analysis are the 889 for whom we had data for both percentage body fat and diet at 4–6 years of age. Characteristics of the cohort are shown in Table 1. About half (48 %) were boys; mean (sd) percentage body fat, weight-for-age *Z*-score and height-for-age *Z*-score was 15·4 (4·8) %, −0·70 (0·85) and −0·54 (0·95), respectively. More than 95 % of the children were enrolled in school. Mean (sd) maternal age at follow-up was 32·3 (5·4) years and caregivers had a mean (sd) of 7·4 (3·7) years of education. When we compared the analytic sample with those lost to follow-up from the main study, there were no significant differences in any of the baseline characteristics (see online supplementary material, Supplemental Table S1).

**Table 1:** Summary statistics for child and caregiver variables in the International Lipid-Based Nutrient Supplements (iLiNS)-DYAD-Ghana trial follow-up at child age 4–6 years

Variable	*N*	Mean or *n*	sd or %	Minimum–maximum
Child characteristics				
Fat mass (%), mean and sd	889	15·4	4·8	0·4–33·5
Weight (kg), mean and sd	885	16·6	2·2	10·1–29·9
Height (cm), mean and sd	885	106·5	5·5	85·0–126·0
BMI-for-age *Z*-score, mean and sd	884	−0·56	0·82	−3·27–2·64
Overweight/obese†, *n* and %	884	23	2·6	–║
Physical activity (vector magnitude count), mean and sd	328	1384	243	816–2066
Sex = male, *n* and %	888	427	48·1	–║
School attendance, *n* and %	885	853	96·4	–║
Fruit consumption (no. of times/week), mean and sd	889	4·0	4·3	0–31
Vegetable consumption‡ (no. of times/week), mean and sd	889	26·8	11·7	4–79
Vegetable consumption§ (no. of times/week), mean and sd	889	4·8	4·9	0–37
Sweet beverages (no. of times/week), mean and sd	889	6·6	5·5	0–36
Sweet snacks (no. of times/week), mean and sd	889	9·2	6·4	0–34
Maternal characteristics				
Maternal BMI (kg/m^2^), mean and sd	832	26·8	5·3	15·9–51·6
Household asset score (standardized), mean and sd	889	0·00	0·99	−1·92–3·15
Maternal education (years), mean and sd	888	7·4	3·7	0·0–16·0
Education ≥ 12 years, *n* and %	888	116	13·1	–║
Maternal age (years), mean and sd	889	32·3	5·4	22·5–49·8
Nulliparous at enrolment, *n* and %	889	286	32·2	–║

† Overweight/obesity defined as BMI-for-age *Z*-score > +1.

‡ Vegetable consumption including consumption of tomatoes and onions.

§ Vegetable consumption excluding consumption of tomatoes and onions.

║ Categorical variable, no minimum–maximum assessed.

Table 1 also shows the mean number of times that selected food items were reportedly consumed during the 7 d before the interview, as follows: 6·6 sweet beverages, 9·2 sweet snacks, 4·0 fruits, 26·8 vegetables (including tomatoes and onions) and 4·8 vegetables (excluding tomatoes and onions). We identified two major dietary patterns from the thirty-one food/beverage items in the questionnaire. Rotated factor patterns are presented in the online supplementary material, Supplemental Table S2. Dietary pattern 1 accounted for 50 % of the variance and consisted of sweet drinks, sweet snacks and eggs (sodas; sweet chocolate and malt drinks; fruit and flavoured drinks; Fanyogo (a brand of frozen yoghurt); sugarcane, sweets and candied coconut; sweet pastries; eggs). Dietary pattern 1 was labelled a snacking pattern because all the items that loaded on pattern 1 except eggs are generally consumed as snacks in this setting. Dietary pattern 2 represented cooked foods (tomatoes and onions consumed in cooked soups, stews and dishes) and accounted for 30 % of the variance. All items in each pattern were positively associated with the pattern.

The mean (sd) vector magnitude counts/min (total physical activity) in this cohort was 1384 (243) and the median (interquartile range) amount of time spent in moderate/vigorous physical activity was 41·6 (27·4) min/d, which represents 5·1 % of total physical activity.

The associations of the child and maternal factors with child body fatness, adjusted only for child age, are shown in Table 2. In these minimally adjusted regression models, percentage body fat was positively associated with maternal BMI (standardized *β* (95 % CI) = 0·10 (0·04, 0·16); *P* = 0·003) and negatively associated with physical activity (standardized *β* (95 % CI) = −0·15 (−0·25, −0·05); *P* = 0·003) but not associated with either dietary pattern 1 (snacking pattern; standardized *β* (95 % CI) = 0·06 (0·00, 0·12); *P* = 0·103) or dietary pattern 2 (cooked foods pattern; standardized *β* (95 % CI) = −0·05 (−0·19, 0·09); *P* = 0·474). We observed a gender difference in percentage body fat and physical activity: boys were significantly more active (boys *v.* girls, 1470 *v.* 1314 mean vector magnitude count/min; *P* < 0·0001) and had significantly lower percentage body fat (13·8 *v.* 16·9 %; *P* < 0·0001) than girls. The only other maternal factor related to child body fatness was maternal parity: children of mothers who were nulliparous at enrolment were leaner than children of multiparous mothers. Further adjustment for sex and intervention group for the relationships shown in Table 2 did not change the estimates except for physical activity, in which case the magnitude of the coefficient was reduced and the relationship became non-significant (standardized *β* (95 % CI) = −0·05 (−0·15, 0·05); *P* = 0·300).

**Table 2:** Associations of maternal and child factors with child percentage body fat in the International Lipid-Based Nutrient Supplements (iLiNS)-DYAD-Ghana trial follow-up at child age 4–6 years

Predictor	Percentage body fat	
	*β*	95 % CI
Child factors		
Physical activity	−0·15	−0·25, −0·05
Dietary pattern 1	0·06	0·00, 0·12
Dietary pattern 2	−0·05	−0·19, 0·09
Child school attendance	0·01	−0·34, 0·36
Child sex	−0·63	−0·75, −0·51
Maternal factors		
Maternal BMI	0·10	0·04, 0·16
Maternal age	0·04	−0·02, 0·10
Nulliparous at enrolment	−0·18	−0·32, −0·04
Maternal education	−0·04	−0·10, 0·02
Household asset score	0·05	−0·01, 0·11

Variables and the outcome were standardized to the mean and sd of the population. All models adjusted for child age. Child sex is coded as 1 = male, 0 = female. The *β* coefficients were obtained from a regression model (SAS PROC REG).

Table 3 shows the associations (minimally adjusted) among the various maternal and child factors considered for inclusion in the path analysis. Both of the child dietary patterns were positively associated with maternal education, and dietary pattern 1 (the snacking pattern) was also positively associated with maternal BMI and household asset score. Maternal education and household asset score were positively associated with maternal BMI.

**Table 3:** Regression coefficients for the associations among predictors (standardized variables) in the conceptual model in the International Lipid-Based Nutrient Supplements (iLiNS)-DYAD-Ghana trial follow-up at child age 4–6 years

	Dietary pattern 1		Dietary pattern 2 <median or ≥median		Physical activity		Maternal BMI		Nulliparous (yes or no)	
	*β*	95 % CI	OR	95 % CI	*β*	95 % CI	*β*	95 % CI	OR	95 % CI
Household asset score	0·19	0·13, 0·25	1·12	0·97, 1·28	–		0·17	0·11, 0·23	–	
Maternal education	0·13	0·07, 0·19	1·20	1·04, 1·37	–		0·07	0·01, 0·13	–	
Maternal BMI	0·15	0·09, 0·21	1·03	0·90, 1·18	–		–		–	
Maternal age	0·04	−0·02, 0·10	1·05	0·92, 1·21	–		–		4·31	4·21, 4·40
Nulliparous at enrolment (yes or no)	0·22	0·08, 0·36	1·31	1·00, 1·72	–		–		–	
School attendance (yes or no)	0·19	−0·14, 0·52	1·51	0·73, 3·11	0·31	−0·28, 0·90	–		–	

Variables were standardized to the mean and sd of the population. The *β* coefficients were obtained from bivariate models adjusted for child age.

**Fig. 3:**
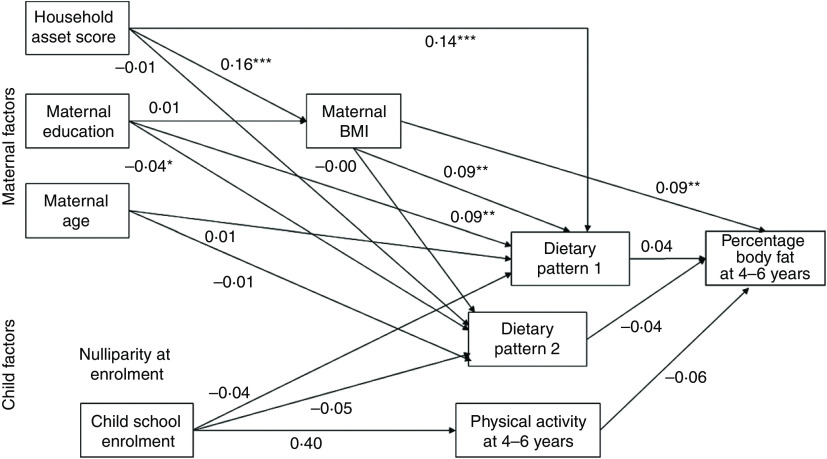
Final path model of the maternal and child factors related to percentage body fat at 4–6 years in the International Lipid-Based Nutrient Supplements (iLiNS)-DYAD-Ghana trial follow-up using structural equation modelling. Numbers in the model are standardized *β* coefficients obtained by including all variables in the model and not from individual regression models. Additionally, the model is adjusted for child age, child sex and intervention group. We did not include nulliparous at enrolment in the final model because maternal age and being nulliparous at enrolment were strongly correlated (*r* = −0·49, *P* < 0·0001). *P < 0·05, **P < 0·01, ***P < 0.001

In a path analysis (fully adjusted model) based on the conceptual model (Fig. 2), only maternal BMI remained significantly related to child percentage body fat (Fig. 3). Maternal BMI, maternal education and household asset score were all positively related to dietary pattern 1 (the snacking pattern). Maternal education remained significantly related to dietary pattern 2 (the cooked foods pattern). However, while maternal education was positively related to dietary pattern 2 in regression analysis, it was negatively related to pattern 2 in the path analysis. The only factor related to maternal BMI in the final path model was household asset score.

## Discussion

In the present study of 4–6-year-old Ghanaian children, maternal BMI was the key factor associated with child percentage body fat. Maternal BMI was also positively related to the snacking dietary pattern, but neither of the two dietary patterns was significantly associated with child percentage body fat. When adjusting only for child age, child physical activity was inversely associated with percentage body fat, but this relationship became non-significant when adjusting for child sex in the fully adjusted analysis because boys had higher physical activity and lower percentage body fat.

The positive association observed between maternal BMI and child percentage body fat has also been reported by others^(^^21^^)^. We cannot disentangle the effects of pre-pregnancy/prenatal BMI from the effects of concurrent BMI, as they are highly correlated (correlation coefficient of 0·86 in our sample). There is evidence for an association of offspring body fat with maternal pre-pregnancy BMI^(^^22^^)^. It is likely that our results reflect a combination of prenatal effects (*in utero* ‘programming’) and postnatal effects due to a shared environment. Positive associations between maternal dietary patterns and child dietary patterns^(^^23^^,^^24^^)^, and between parental physical activity and child physical activity^(^^25^^,^^26^^)^, have been reported.

We did not find a significant association between the two dietary patterns we identified and percentage body fat. Some studies^(^^5^^,^^27^^–^^29^^)^ have reported an association between certain dietary patterns and measures of body fat in children. In the Avon Longitudinal Study of Parents and Children (ALSPAC), an energy-dense, high-fat, low-fibre dietary pattern at 7, 10 and 13 years of age was positively associated with subsequent fat mass index at 11, 13 and 15 years of age^(^^5^^)^. Similar associations have also been reported in the ALSPAC cohort, whereby the energy-dense, high-fat, low-fibre dietary pattern scores at 5 and 7 years of age were associated with increased adiposity at 7 and 9 years of age^(^^27^^)^ and a junk food dietary pattern at age 3 years was associated with increased risk of obesity at 7 years of age^(^^28^^)^. In the Portugal Generation XXI cohort, an energy-dense foods dietary pattern at 4 years was associated with fat mass index at 7 years in girls but not boys^(^^29^^)^. One possible reason for not detecting such an association in our study is that the overweight/obesity prevalence (BMI-for-age *Z*-score > +1) in our cohort was only 2·6 %, whereas it was at least 10 % at all time points in the ALSPAC cohort. Also, we examined the relationship between concurrent diet and percentage body fat, whereas the ALSPAC study examined the association between a dietary pattern identified at an earlier age and body fat measures at a later age. Overweight prevalence will likely increase if there are no interventions and dietary patterns also usually track into later life^(^^27^^,^^29^^,^^30^^)^. Thus, we may begin to see harmful effects of the snacking pattern in our cohort later even though it is not evident now. We also note that our dietary data collection tool was not designed specifically to capture energy density of the diet. Lastly, the positive association between the ‘junk food’ dietary pattern and body fat described above was observed in high-income populations, which may have even greater access to these foods compared with the Ghana cohort.

The association between physical activity and percentage body fat in our data was negative and significant in regression analysis adjusting only for child age; however, the relationship became non-significant when we adjusted additionally for child sex. We observed significantly greater physical activity among boys than among girls at 4–6 years of age, which is similar to findings in a Swedish cohort of 4·5-year-old children^(^^21^^)^. In a meta-analysis, there was a weak non-significant inverse association between physical activity and body fat^(^^31^^)^. Three of the six studies in the meta-analysis adjusted for sex, which may attenuate the relationship between physical activity and body fat if boys are leaner and more active compared with girls. Two studies included in the meta-analysis^(^^32^^,^^33^^)^ reported information on gender differences in physical activity, with both showing greater physical activity in boys compared with girls. One of those studies^(^^30^^)^ also examined gender differences in body composition, showing greater lean tissue mass in boys compared with girls but no difference in fat mass or percentage body fat. Evidence published since the meta-analysis regarding the association between physical activity and body fat in children has been mixed, with some studies showing no association^(^^34^^)^ and others showing significant negative associations^(^^5^^,^^35^^,^^36^^)^. These studies used different measures of physical activity and adiposity which may contribute to the variation in findings. Inconsistency in results may also be related to the use of longitudinal *v.* cross-sectional approaches, i.e. some studies examined change in body fat or BMI (by controlling for baseline adiposity) whereas others examined only the concurrent relationship between physical activity and body composition (as in our study).

Although it has been reported that caregiver education is associated with healthier eating patterns^(^^23^^,^^27^^,^^37^^,^^38^^)^ in higher-income populations, we found that maternal education was positively associated with the snacking pattern and negatively associated with the cooked foods dietary pattern when controlling for all the other variables in the final model. The mean number of years of maternal education in our study was 7 years and only 13·1 % had an educational attainment of 12 years or more, whereas the association reported in the other studies was based on comparing those with a university education with those having less education. We also observed a positive relationship between household asset score and both the child’s snacking pattern and maternal BMI. Amugsi *et al.*^(^^39^^)^ found a similar association between household wealth and maternal BMI using Ghana DHS data. Our cohort is a semi-urban population in a country experiencing the nutrition transition; increasing incomes therefore mean more access to diverse snack foods and sweetened beverages. We observed a positive relationship between maternal BMI and both child body fat and the snacking dietary pattern, but no significant association between the snacking pattern and child body fat. The potentially negative effects of a diet dominated by snack foods of low nutritional value, particularly those with added sugar, may not be evident yet and thus further follow-up of this cohort would be useful.

The strengths of our study include the large sample size, the use of the ^2^H dilution method to assess body composition, and the inclusion of both dietary factors and physical activity as potential predictors of child percentage body fat. We compared the children in the analysis with those who were lost to follow-up and there were no differences in any of the baseline characteristics, indicating no attrition bias and suggesting that our findings are generalizable to the study population enrolled. A limitation is the use of an FFQ that was primarily designed to capture consumption of sugary foods and beverages and not specifically designed to capture all of the dietary characteristics that might be related to body composition. In addition, the recall period was the 7 d before the interview which may not be reflective of the usual consumption habits of participants. The questionnaire was not validated in our study population; however, items included in the questionnaire were based on a pilot study conducted before the follow-up began. The use of factor analysis to create the dietary pattern factors takes advantage of correlation between dietary variables, but a potential limitation of this approach is that it is data-driven and the factors generated may not always be easy to interpret. In this case the patterns generated were interpretable. However, we did not assess maternal dietary patterns, which may have given further insight into the food behaviour environment. Lastly, because of the cross-sectional nature of the associations observed, we are unable to determine the direction of these associations.

## Conclusion

In conclusion, in the present study of 4–6-year-old Ghanaian children with a low prevalence of overweight or obesity, we identified two major dietary patterns, a snacking pattern and a pattern depicting cooked foods, neither of which was associated with child percentage body fat. Maternal BMI was positively associated with child body fat while physical activity was inversely associated with body fat, an association that became non-significant when we adjusted for child sex. We speculate that maternal lifestyle changes may be important in reducing the risk of child overweight/obesity in this population, given the potential influence of the mother’s body composition during pregnancy and considering that her diet and physical activity may affect the child’s food and activity patterns. In addition, improving child physical activity, especially among girls, may be important for reducing the risk of child overweight.

## Supplementary material

For supplementary material accompanying this paper visit https://doi.org/10.1017/S1368980019001745. click here to view supplementary material
