# Supplementary material for: Maternal and child factors associated with child body fatness in a Ghanaian cohort
Source: Public Health Nutr. 2019 Jul 25;23(2):309–18. doi: 10.1017/S1368980019001745 (PMC6988376; doi:10.1017/S1368980019001745)
Supplement: Supplementary file 1 [file S1368980019001745sup001.doc]

**Supplementary Table 1: Background characteristics of women and children included in the analysis and those not included in the International Lipid-Based Nutrient Supplements (iLiNS)-DYAD Ghana trial follow-up at child age 4-6 y**

|  | **In the follow-up**  **[n=889]** | |  | **Not in the follow-up**  **[n=431]** | |  | **p-value** |
| --- | --- | --- | --- | --- | --- | --- | --- |
| **Variable** | **x̅** | **SD** |  | **x̅** | **SD** |  |  |
| **Maternal Characteristics** | |  |  |  |  |  |  |
| Age (y) | 26.8 | 5.4 |  | 26.4 | 5.7 |  | 0.096 |
| Gestational age at enrolment (wk) | 16.1 | 3.3 |  | 16.0 | 3.3 |  | 0.960 |
| Years of formal education (y) | 7.6 | 3.5 |  | 7.6 | 3.9 |  | 0.991 |
| Married or Cohabiting (% [n]) | 93.4 | 830/889 |  | 91.4 | 393/430 |  | 0.197 |
| Household asset score (standardized) | 0.01 | 0.97 |  | -0.01 | 1.1 |  | 0.747 |
| Nulliparous at enrollment (% [n]) | 32.2 | 286/889 |  | 37.2 | 160/430 |  | 0.070 |
| Weight (kg) | 62.0 | 12.1 |  | 61.6 | 11.5 |  | 0.520 |
| Height (cm) | 159.0 | 5.7 |  | 158.5 | 5.7 |  | 0.135 |
| BMI (kg/m^2^) | 24.5 | 4.5 |  | 24.5 | 4.2 |  | 0.814 |
| Overweight (BMI ≥ 25) (% [n]) | 31.7 | 277/873 |  | 31.3 | 131/418 |  | 0.394 |
|  |  |  |  |  |  |  |  |
| **Child Characteristics** |  |  |  |  |  |  |  |
| Sex of child, boys (%) | 48.1 | – |  | 51.3 |  |  | 0.311 |
| Birth weight (kg) | 3.0 | 0.4 |  | 3.0 | 0.4 |  | 0.586 |

BMI, Body Mass Index

Values in the table indicate mean, SD unless otherwise indicated

**Supplementary Table 2: Rotated dietary patterns identified from factor analysis.**

| **Variable** | **Factor 1** | **Factor 2** |
| --- | --- | --- |
| Sweet teas | 0.15 | 0.03 |
| Sodas | **0.43** | -0.04 |
| Chocolate and malt drinks | **0.39** | -0.05 |
| Fruit/flavoured drinks | **0.42** | 0.00 |
| Sweetened milk beverages | 0.33 | 0.01 |
| Dairy (milk) | 0.13 | 0.01 |
| Yoghurt drinks | 0.17 | 0.02 |
| Fanyogo (brand of frozen yoghurt) | **0.40** | -0.12 |
| Sugarcane, sweets, candied coconut | **0.39** | 0.0 |
| Sweet pastries (fried/baked goods) | **0.38** | 0.06 |
| Savoury snacks (fried/baked goods) | 0.32 | -0.03 |
| Sweet groundnut snacks e.g. brittle, batter coated nuts | 0.22 | 0.08 |
| Savoury groundnut snacks e.g. roasted nuts | 0.13 | -0.01 |
| Vitamin A rich fruits | 0.03 | 0.08 |
| Other fruits | 0.32 | -0.04 |
| Dark green leafy vegetables | 0.05 | 0.07 |
| Soups, stews, dishes made with tomatoes | 0.07 | **0.80** |
| Soups, stews, dishes made with onions | 0.05 | **0.95** |
| Vitamin A rich vegetables eaten in soup, stews or dish | 0.04 | 0.02 |
| Vitamin A rich vegetables eaten as is | 0.10 | -0.02 |
| Other vegetables in soups, stews or dishes | -0.05 | 0.17 |
| Melon seeds in soups, stews or dishes | 0.18 | 0.01 |
| Vegetables eaten as is | 0.07 | 0.02 |
| Dishes or soup/stews made with beans | 0.16 | 0.04 |
| Dishes or soup made with groundnut | 0.05 | 0.06 |
| Fish | -0.10 | 0.30 |
| Seafood | 0.10 | 0.01 |
| Meats and poultry | 0.17 | 0.06 |
| Cow skin | 0.05 | -0.07 |
| Eggs | **0.41** | -0.06 |
| Cooking oils | -0.12 | 0.08 |

Factor loading in bold: ≥0.35
